# Electronic prescription as a driver for digitalization in Finnish pharmacies

**DOI:** 10.1186/s12913-021-07003-0

**Published:** 2021-09-27

**Authors:** Teijo Peltoniemi, Reima Suomi, Sirpa Peura, Markus N. Y. Lähteenoja

**Affiliations:** 1 University of Turku, 20014 Turun Yliopisto, Turku, Finland; 2 Lähiapteekki Syke, 15140 Lahti, Finland

**Keywords:** Digitalization, Community pharmacy, Dispensing process, Electronic prescription, Sociotechnical system

## Abstract

**Background:**

Finnish community pharmacies have undergone digitalization during the past decade. The introduction of the electronic prescription has had a significant impact on pharmacy workflows, such as the dispensing process. This inevitably has significant sociotechnical implications. We examine the impact of digitalization on the dispensing process and the sociotechnical orientation of a pharmacy.

**Methods:**

We utilize data collected in customer service situations in Finnish community pharmacies at two points in time: in the traditional workflow, when electronic prescriptions were not in use, and in the new direct dispensing workflow, which is the usual delivery model in the case of electronic prescriptions. We analyze this data in terms of changes in workflow efficiency. We also draw on existing literature to build a conceptual model for digitalization in the pharmacy sector from a sociotechnical standpoint.

**Results:**

In the Finnish environment, the results, based on our study sample, show that with electronic prescriptions and the direct dispensing model, the delivery time for a single medication over the counter was reduced by 13%. The results also indicate that the process has become more predictable, as the variation in terms of the workflow lead time has decreased.

**Conclusions:**

The results indicate that the dispensing process has become more efficient in terms of time and throughput as well as more technically oriented and predictable. From a sociotechnical perspective, the results indicate that the technical subsystem has strengthened, and pharmacies have adapted to the new technology in the dispensing process.

## Background

### Introduction

The pharmacy sector is not immune to pervasive digitalization. Innovations, such as robots, have disrupted the pharmacy practice [1]. One major element affecting this sector is the electronic prescription, or ePrescription, which can be defined as “the use of computing devices to enter, modify, review, and output or communicate drug prescriptions” [2]. Electronic prescribing is argued to have potential to streamline and improve medicine dispensing [3–5]. The potential benefits include a decrease in medication errors [6, 7] and efficient identification of adverse effects [8].

ePrescriptions have been widely adopted in Finland [9]. Another significant change in the Finnish community pharmacy sector is the introduction of the direct dispensing model – a model that streamlines the prescription-only (PO) customer service process with the support of new technology. In this article, we examine what these changes mean for Finnish pharmacies and discuss the impact from a sociotechnical perspective.

### ePrescription and the direct dispensing model

The Finnish pharmacy sector has undergone significant changes in recent years. Electronic prescription has been widely adopted in Finland since 2010, and it became the sole prescription method in 2017 [9–11]. The use of electronic prescription and the related centrally governed databases are mandated by law in Finland [12]. The Finnish Patient Data Repository (KanTa) is a centralized database containing personal healthcare records with a separate partition for medicine prescriptions [13].

The Finnish KanTa is a wide ecosystem digitally integrating Finnish healthcare service providers and pharmacies through a centralized data source containing records for each Finnish citizen [14]. It is considered a pivotal integration mechanism within the medicine supply chain. Physicians can, e.g., investigate previous prescriptions ordered by any healthcare service provider while prescribing. Pharmacists access the database to fetch prescriptions when dispensing the medicine, and the prescription can be dispensed in any pharmacy, in contrast to implementation in some countries, where the prescription is transmitted only to a selected pharmacy.

The benefits of this complete digitalization of the medicine prescribing process include avoidance of overlapping medication [15], a clear overview of the total patient medication [16], and uniform medicine information between health care units and pharmacies [9]. According to Aanestad et al. [17], ePrescription systems have the potential to improve control of medicine expenses and related performance management. This type of wide-scale centralized ePrescription solution is a rarity worldwide, and even in Europe [18].

ePrescriptions were a key trigger of the transformation of the pharmacy dispensing process; the pharmacists collect, handle and finally sign off on the dispensed medicine in the centralized online ePrescription service without first copying the content of the prescription to the pharmacy IT system [19, 20]. This means that medicine dispensing had to transform in all pharmacies from a workflow that we call the “traditional dispensing model” to a model that we call the “direct dispensing model”.

**Fig. 1:**
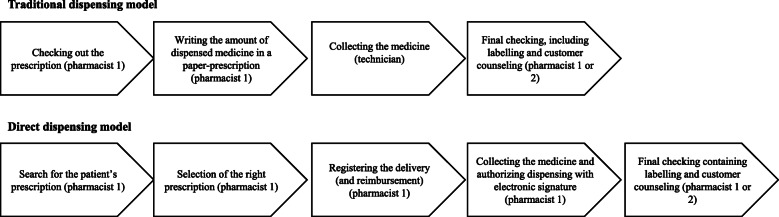
Traditional and direct dispensing models

Several differences exist between the workflows of these two dispensing models. The main difference – from the customer point of view – is that in the direct dispensing model, the customer is served in an uninterrupted episode by a single pharmacist handling prescriptions through an IT system, while in the traditional dispensing model, there are several pharmacists and technical workers involved in the workflow. The two delivery models are depicted in Fig. 1.

The direct dispensing may be divided into two different models, the older one with paper prescriptions and the present one with ePrescriptions. In the older model, the paper prescriptions are copied into the pharmacy IT system, which collects the data of the dispensed prescriptions, maintains documentation and includes tools for reimbursement calculation. The work is done in face-to-face interaction with the customer, by one pharmacist. For the sake of clarity, we will refer to these three different models as mode 1, mode 2 and mode 3, as follows:

- Mode 1: Traditional dispensing model with paper prescriptions;
- Mode 2: Direct dispensing model with paper prescriptions;
- Mode 3: Direct dispensing model with ePrescriptions.

Where electronic prescription is used, the pharmacist is supported by a view of all the prescriptions of the customer. Finding the right prescription may be time-consuming when the customer has multiple prescriptions and prescription data from a long time period. As the prescriptions are already in electronic form, a pharmacist does not have to copy the data to the pharmacy IT system. The critical and most value adding phases in the process regarding quality are the professional checking of the prescription content and the implementation of the reimbursement rules. After that, the medicine can be collected and dispensed.

In the traditional dispensing model, the originality of the prescription must be evaluated, and this task is not needed in the case of electronic prescription. Additionally, copying the prescription content to the pharmacy IT system is mandatory. In the traditional dispensing model, the customer session was often broken into many service episodes, which engaged several employees including pharmacy technicians.

### The sociotechnical model

The sociotechnical system approach examines information systems from the point of view of both social and technical subsystems [21]. It focuses especially on the interaction between these subsystems. According to Berg et al. [22] p. 297, the sociotechnical approach seeks to “*increase our understanding of how information systems or novel electronic communication techniques are developed, introduced and become a part of social practice*”.

Relating to the pharmacy domain, sociotechnical theory has been utilized often in relation to ePrescription. For example, Aarts [23] explains that prescribing occurs in social settings and that related technologies influence the behaviors of those involved. Prescribing is sociotechnically a complex event, which completes the interaction between a physician and a patient. The prescription as a technology can “nudge” the related stakeholders in a certain direction – e.g., ePrescription increases transparency, which may steer patients’ and physicians’ behavior (previous prescriptions cannot be hidden). Understanding these sociotechnical aspects, as Aarts [23] argues, is crucial to the success of the adoption of ePrescription.

To summarize a few other sociotechnical studies, it is, for example, argued that new types of medication error might occur in connection with the interplay of several electronic systems [5, 24] or pharmacists may simply exhibit mental resistance towards new kinds of systems [25].

Another line of discussion involves deskilling workers. It is argued that technology will change professional boundaries and identities, including in pharmacy settings [1, 26]. Whereas some pharmacy workers may benefit from the deployment of a new technologies, some will be affected detrimentally, as they lose control of their work and their incentives to develop their professional skills. The same applies to ePrescription; it has a sociotechnical impact through changing organizational relationships and tasks, which may entail deskilling for some and reskilling for others [26].

To counterbalance an overly deterministic view of technology, recently, the concept of “affordance” has received much attention in information systems and marketing research. According to the concept of affordance, technology provides – i.e. affords – opportunities and risks based on the materiality of the technological artefacts [27]. It is up to the social subsystem to utilize or avoid them. Technology shapes the social rather than other way around, which, in practice, may lead to novel forms of usage, which may be different than those the solution was originally designed for (consider, for example, how firms have begun to use Twitter for marketing, which is not the intended model for using it). Petrakaki et al. [28] discuss how the affordance of ePrescription as a technology can construct risks, such as blame-shifting, which takes place when responsibility is displaced – in this case, it entails widening the jurisdiction of pharmacy staff. This happens through a social process, rather than being determined by the technology.

Harvey, Avery, Waring and Barber [29] researched the sociotechnical impact of ePrescription in community pharmacies in the UK in relation to the upcoming release of a national ePrescription service. They discovered that pharmacies can be divided into three categories based on the sociotechnical orientation: technically oriented, improvising and socially oriented.

According to Harvey et al. [29], technically oriented pharmacies have tightly governed workflows and typically utilize technologies such as robots, computerized prescription order entry systems and pharmacy manager systems. In their study, they measured dispensing lead times in these three types of pharmacies. The dispensing lead times were reported as being 2–5 min. At the other end of the spectrum, they found socially oriented pharmacies that based dispensing on adaptive and customized workflows with few technical aids. The workflows were, however, found to be effective, and the dispensing journey took 3–8 min on average. In the middle were the improvising type of pharmacies, which were not oriented in either direction and were often troubled by disorganized and unpredictable workflows. The dispensing lead times with improvising pharmacies were reported as being 10–14 min.

### The aim of this study

Whereas ePrescription implementations in different environments have been studied widely, research on the ePrescription system is scarce in terms of its impact on pharmacy workflows, practices, and digital transformation, which is also true in the Finnish setting. The dispensing process ending in the handing of the medicine to the customer is the “moment of truth” in the prescribing process, and has a crucial impact on the further acts of the customer. It is the last point at which it is typically possible to give personal guidance about the medicine to the customer, and it may even be decisive in the customer’s decision to consume or not consume the prescribed medicines. Therefore, the dispensing process deserves careful attention from researchers.

Given that the Finnish ePrescription system is relatively developed and widely adopted, studying it can give valuable input to countries where the development is in more nascent phases. We utilize a sociotechnical lens to understand the disruption these new technologies may generate for pharmacy staff. The particular research questions are as follows:

- *RQ1: How ePrescription has changed the dispensing workflow;*
- *RQ2: What kind of sociotechnical impact this has.*

## Methods

Broadly, our study falls into the area of business process analysis and management [30]. Time management and throughput/lead time reduction are key touchpoints in process management [31], especially in human service [32], as well as healthcare settings [33–35].

In our research, the needed interaction time for dispensing one patient’s medicines for one prescription at the counter of a community pharmacy was measured. The process started with the customer arriving at the counter and ended with handing out the medicine to the patient.

Measurement occurred in a discrete setting at a distance from the service counter, but it was allowed to differentiate between the different phases of the dispensing. The phases separated and recorded are those presented in Fig. 1. The phases are based on the official, obligatory guidance by the Finnish Medicine Acency [36]. Payment for the medicine was not included in the measured process, as in many cases and pharmacies, payment is made at a separate desk.

Measurements took place at two time points: the first was in the year 2006,^1^ when paper prescription was the only prescription type, but when both Mode 1 (the traditional dispensing model) and Mode 2 (the direct dispensing model) were in use.^2^ In the year 2012, a second measurement took place. At this time, Mode 3 (direct dispensing with electronic prescription) was also in use, and the direct dispensing model was the only workflow type used.

Together measurements happened in five middle-sized community pharmacies in Helsinki metropolitan area. There is no indication that these pharmacies and their customers would not be representative of the whole Finnish pharmacy field. The pharmacies and persons measured were not the same in the years 2006 and 2012, and the practical measurements were also done by different researchers. However, the measurement process guidance came from the same persons (co-authors SP and RS of this article) at both measurement times, allowing for direct control of the measurement performance.

The researchers performing the measurement were given guidance on how to perform the measurement in the form of a measurement protocol guide. Measurement continued until the measurement team felt that no new types of service encounter situations emerged and that the situations were repetitions of earlier medicine dispensing situations. Effort was made to eliminate the differences in transactions at different timepoints during the week by performing measurements at different weekdays.

Each service encounter was measured by one person. After the measurement sessions, extremely short or long service episodes were discussed by the research team present. In this discussion, measurement items, in the case of which it was interpreted that some external event, such as third person entering the service episode, or some specific issue with the medicine (such as not having the medicine in stock), or technical issue with the used (information) systems, affected the time needed, were eliminated from the data. In the same way cases where it was not possible to clearly differentiate between the different phases of our dispensing models were eliminated from the data. So there are no missing values in the data. Each service episode was unique (client, pharmacy worker, medicine dispensed), and they cannot be compared with each other.

Ethical permission for the study was granted by the Finnish Association of Pharmacies. It is important to note that the researchers did not have any possibility of identifying the customers served and that the screen view of the workstation was viewed from a distance that allowed the identification of the work phase (through identification of the screen layout), but not the identification of any more detailed data, such as the customer identification or the identification of the medicines dispensed. The screening of the process happened from such a distance that it was not possible for the customers to associate the activity with their service encounter situation. The pharmacy workers did not know exactly when their activity was monitored.

Permission for the follow-up on worker performance was acquired from the workers before starting the survey. It was clear to all that the data were collected only for scientific purposes and that the purpose of surveillance was not any personal assessment of the workers or delivery of any rewards or punishment of any kind.

The measured data were entered into Excel sheets immediately at the time point of observation. Excel was a versatile enough tool to enable further analysis of the data, as presented in this article. Cases in which the process was disturbed by an external factor (for example, a third person entering the service delivery situation or the abandonment of the service transaction by the customer) were excluded from the data, as well as episodes where the observers felt that they could not clearly separate the different phases of medicine delivery.

In the year 2006 research, 249 customer sessions and 379 prescriptions were reported at three pharmacies. The focus was on the effects of technology on the pharmacy workflow, and our unit of analysis was the time needed to handle one customer prescription. In the year 2012 study, the delivery of 848 prescriptions in 470 customer sessions were monitored and reported. Data collection took place in five pharmacies, which were large or middle-sized pharmacies. Two different pharmacy systems were used.

The customer situations were different - a customer fetching a new medicine for the first time was regarded as a standard case. The situation is, however, different in the case of a renewed prescription: the customer is already familiar with the medicine, and for example, the need for counselling may be lower. Furthermore, one customer session could involve one or many prescriptions. The customer might or might not carry with him/her the information sheet for the medicine to be dispensed, either provided by the prescribing medical doctor or printed out from the KanTa system by the customer. The details of the observed dispensing processes are outlined in Table 1.

**Table 1:** Number of prescription delivery processes analyzed in the years 2006 and 2012

	Mode 1–2006	Mode 2–2006	Mode 2–2012	Mode 3–2012
Customer sessions	126	123	347	162
Number of prescriptions handled	189	190	573	275
Number of packets delivered	210	234	704	322

## Results

The data indicate that changes occurred between 2006 and 2012, and it seems that the process of dispensing became faster in Finnish community pharmacies after 2006. Table 2 contains a summary of the data associated with the PO medicine dispensing lead times. The results show that with the electronic prescription, the average dispensing time for a single prescription (Mode 3) in 2012 was 13.1% (22 s) shorter than that of the direct dispensing model with paper prescriptions (Mode 2) in 2006.

**Table 2:** Total delivery times for different types of prescriptions in the years 2006 and 2012

	Mode 1–2006	Mode 2–2006	Mode 2–2012	Mode 3–2012
median time	2 min 48 s	2 min 38 s	1 min 43 s	2 min 4 s
average time	3 min 26 s	2 min 48 s	1 min 58 s	2 min 26 s
standard deviation	1 min 50 s	1 min 34 s	57 s	1 min 17 s
minimum time	1 min 4 s	34 s	32 s	36 s
maximum time	12 min 55 s	12 min 40 s	6 min 22 s	9 min 15 s

The saved time was greater in the case of Mode 1 (traditional dispensing with paper prescriptions), where the average time for dispensing was reduced by 29.1% (60 s) compared to Mode 3. However, Mode 2 (direct dispensing with paper prescriptions) in 2012 was 19.2% (28 s) faster on average than Mode 3 (direct dispensing with ePrescriptions). There may be various reasons for this: for example, during the time of the data collection, ePrescriptions had only just been adopted, and the supporting IT infrastructure was still being developed and was in an immature state. However, the paper prescription processing functionalities were in an established and therefore optimized state.

In addition to the decreased median and average times, we find a reduction in the maximum time for dispensing to take place. In 2006, the maximum time recorded with Mode 1 was 12 min and 55 s, and that with Mode 2 was 12 min and 40 s, whereas in 2012, these times were 6 min and 22 s (Mode 2) and 9 min and 15 s (Mode 3). Also, the deviation was more limited: in 2006, the standard deviation with Mode 2 was 1 min and 34 s, whereas in 2012, it was 57 s.

When comparing ePrescription and paper prescription and the lead times of individual tasks in the direct dispensing process, as depicted in Tables 3 and 4, we can conclude that searching and signing tasks take extra time, which makes the ePrescription-based process slower. This may imply that the user interface and integration of the pharmacy and ePrescription systems still need to mature. This is supported by the fact that the standard deviation and maximum time of the process is greater with ePrescription; for example, addressing technical glitches with the systems and their integration takes time. It can also be intuitively inferred that adding new technology with additional process steps will increase the process lead time; users must learn a new system, and it is slower to use in the beginning.

**Table 3:** Results per task for paper prescription (seconds), 2006 results in parentheses

Paper prescription (2006 results in parentheses)					
*n* = 573	Checkout	Marking	Fetching	Final check and counselling	Total time
median time	13.0 (14.0)	40.8 (57)	10.0 (0)	36.0 (60)	102.7 (158)
average time	16.1	45.4 (63)	11.3 (12)	45.0 (68)	117.8 (168)
standard deviation	13.4	27.7	16.2	29.4	56.7
minimum time	2.0	0	0	0	32
maximum time	125.0	291.0	179.0	194.0	382

**Table 4:** Results per task for ePrescription (seconds)

ePrescription							
*n* = 275	Search	Selection	Registering	Signing	Fetching	Final check and counselling	Total time
median time	10.0	14.5	36.6	6.3	10.0	36.0	124.0
average time	14.2	21.8	43.1	6.7	14.9	45.7	146.4
standard deviation	12.8	26.1	33.4	4.8	27.8	32.8	76.8
minimum time	1.0	0.0	3.0	1.0	0.0	5.0	36.0
maximum time	93.0	226.0	350.0	34.0	320.0	283.0	555.0

## Discussion

Considering these results, one possible implication is that the dispensing process and the associated task structure have become more controlled and less improvised. The decreased deviations and maximum time outliers imply that the process has become more stable and predictable, which could be a result of stricter process governance. This could indicate that the given pharmacies have been able to standardize work processes and migrate to the new technology-driven dispensing process (Mode 2 and Mode 3). The process has become faster according to the times recorded for the traditional dispensing model in 2006. The direct dispensing model has become faster as well, although ePrescription has been introduced as an additional application.

From a sociotechnical perspective, the results could be interpreted as indicating that pharmacies have adapted a stronger technical orientation at the cost of social orientation. This also means stricter task structures, which are built around the technical system. As Harvey et al. [29] suggest, socially oriented and improvising pharmacies have the most significant prospects for making productivity gains through adopting ePrescription, since it forces them to streamline their workflows. This seems to be the case with Finnish community pharmacies when examining the efficiency gains obtained by shifting from the traditional to the direct dispensing model. The changes for direct dispensing pharmacies, i.e., technically oriented pharmacies as described by Harvey et al. [29], in adopting ePrescription are less significant, implying that they have successfully integrated ePrescription into their existing professional process and technical sub-system in their sociotechnical environment.

Another sociotechnical impact relates to the nature of the work. Whereas the time was spent in manual tasks related to paper prescriptions, in the current setup with ePrescription, the pharmacy dispensing worker can focus on customer service work. This, of course, may have several implications, such as extending the jurisdiction of pharmacy staff. The data cannot be used to analyze this any further, and to validate this, more research is required.

To conclude the sociotechnical discussion, it can be anticipated that implementing direct dispensing to pharmacies, enabled by electronic prescriptions, will have an impact on the tasks of different groups of pharmacy workers and may also change the composition of pharmacy staff in terms of roles and skillsets [20]. We can observe that between 2002 and 2019, the amount of technical staff did not decrease [37, 38]. If we, however, relate the number of prescriptions dispensed annually to the number of pharmacy staff, it seems that the sector has become more efficient: whereas, in 2002, there were 38.5 million prescriptions processed in Finnish pharmacies, in 2019, the amount had risen to 65.7 million [37–39]. The number of prescriptions is predicted to have grown in 2020 as well [39], and there is no reason visible why the growth would not continue even in the future. The number of pharmacists in Finnish pharmacies grew from 4654 to 5594 between 2002 and 2020, and the number of technical staff grew from 2949 to 3224 [37, 40]. These figures indicate that currently, roughly the same amount of staff process almost twice as many prescriptions than they did in 2002, which also supports the finding that profound changes have occurred in pharmacy workflows.

Hassell et al. [41] report that the workload of pharmacists has increased and that dispensing takes the majority of their time. This is aligned with our finding relating to the time spent counseling with customers: it has not increased. Hassell et al. [41] also conclude that it is difficult to specify what tasks dispensing is comprised of. For example, in our process model counseling cannot be separated from the dispensing process. Counseling can also happen as a simultaneous activity aside other phases of work.

Dispensing has also been investigated from customer waiting time perspective. The model of dispensing process by Alam et al. [42] is aligned with our traditional dispensing model, and it includes the following phases: prescription receiving, checking prescription, order entry, prescription filling, verification and dispensation. In this model the last phase, dispensation, includes also counseling. According to Alam et al. [42] factors impacting the waiting time include work sequencing and problems with the prescription, e.g. the pharmacy staff unable to read the prescription. One of the solutions they suggest is a pharmacy automation system, which corresponds to the direct delivery dispensing process described in this article.

We also acknowledge that our research data represent a past era in the Finnish pharmacy sector, as at present, ePrescription is already an established and thoroughly adopted technology. Observing Finnish community pharmacies today, one can find several digital solutions deployed widely in the field, including, for example, warehouse robotics, automatic pick-up stations and mobile applications for customer service, in addition to e-commerce for medicines. The launch of ePrescription and the direct dispensing model have, however, been significant milestones in the digitalization journey. The automation of the pharmacy workflows requires that prescription, which is a key concept for a pharmacy, is in a digital and highly standardized format. Furthermore, the direct dispensing model has paved the way for launching digital solutions automating pharmacy workflows.

Recently, ePrescription has proven to be a useful tool in combating global pandemics. The COVID-19 outbreak in the spring of 2020 introduced an undesirable phenomenon whereby people began stockpiling medicines [43]. ePrescription in the form in which it is implemented in Finland can manage this through its complete view of prescription histories. Although this applies to prescription medicines, pharmacies have also controlled the buying of over-the-counter medicines. They have, for example, restricted the amount of painkillers that can be bought at the same time, according to guidance issued by the Finnish Medicines Agency (Fimea) [44].

However, perhaps the main benefit of ePrescription during the pandemic is its potential to support remote operations, such as renewing prescriptions without physical visits. The WHO [45] suggests this, and this view is also shared in the field. Indeed, Finnish community pharmacies received a surge in their digital channels - there were 14 times more customers in the online shops than there were before the pandemics [40]. ePrescription supports these types of new operating models, given that it provides a reliable source for authorized prescriptions, which can be transmitted and verified digitally. This aspect of ePrescription makes available another set of novel affordances. Again, this requires a dedicated study, and we are not suggesting any inferences from our historical data.

Another viewpoint is the regulatory environment. The pharmacy sector is a highly regulated field in Finland, as everywhere, and not an ordinary market with ordinary market drivers; one could question whether this transformation would have been as extensive without regulation. Instead, it can be argued that the regulator has nudged the sector to digitalize by mandating the adoption in a top-down manner. Building up the ePrescription system has naturally benefited from several structural aspects present in the Finnish market, such as the unique national identifiers assigned to each citizen and a developed digital identification framework. For policymakers it is also important to understand the various affordances ePrescription provides, such as those relating to online pharmacies. The impact of ePrescription on overall digitalization, as well as the role of regulation, however, requires separate research.

## Conclusions

Based on our study, it seems that the dispensing process became faster after the deployment of ePrescription and the direct dispensing model. There is also an indication that the process has become more predictive and governed. This could be interpreted as indicating that the dispensing process has become more technically oriented and less dependent on human skills and orientation.

Secondly, we argue that ePrescription has been a key driver of digitalization in the Finnish community pharmacy sector, as it has triggered the change to the direct dispensing model and the adoption of other digital solutions in pharmacies.

In terms of limitations, our results are specifically for the Finnish pharmacy system. The implementations of electronic prescriptions vary significantly between countries and even within countries. Even within the same electronic prescription infrastructure, the integration between ePrescription and the pharmacy system can take various forms. In addition to the system integration aspect, the capabilities of pharmacies and individual workers to adapt to electronic prescription vary. Furthermore, the measurement took place when the application of electronic prescription had just started. In Finland, however, according to a recent study, both Finnish pharmacy owners and employed pharmacists define themselves as innovators who are willing to adopt IT innovations [46].

Further research avenues could include studies performing the same measurements as those performed here in different countries as well as in different kinds of pharmacies. The service differences for different customer groups could be analyzed. Adopting the crucial customer viewpoint in the service process by inquiring into customer experiences would add to our understanding of the dispensing process. Distilling service times from system log data would be a convenient option, but in our setting, the implementation of such a system was considered overly complicated, taking into account the current technical status of the systems used.

## Data Availability

The datasets used and/or analyzed during the current study are available from the corresponding author on reasonable request.

## Footnotes

1 Koiranen, M. &J. Sinnemäki, J. (2006). “Reseptitoimitukseen kuluneet ajat suoratoimitus- ja perinteisellä mallilla (Times needed for prescription delivery with the traditional and direct dispensing workflow models).” Unpublished manuscript. Turku School of Economics, 2006.

2 Lähteenoja, M. (2013). “E-reseptin ja paperireseptin toimitusajat suoratoimitusapteekeissa (The delivery lead times of Prescription and paper prescription in direct dispensing pharmacies).” Master’s thesis. University of Turku.
